# A particle-based model for endothelial cell migration under flow conditions

**DOI:** 10.1007/s10237-019-01239-w

**Published:** 2019-10-17

**Authors:** P. S. Zun, A. J. Narracott, P. C. Evans, B. J. M. van Rooij, A. G. Hoekstra

**Affiliations:** 1 Institute for Informatics, Faculty of Science, University of Amsterdam, Amsterdam, The Netherlands; 2 Biomechanics Laboratory, Department of Biomedical Engineering, Erasmus Medical Center, Rotterdam, The Netherlands; 3 National Center for Cognitive Technologies, ITMO University, Saint Petersburg, Russia; 4 Department of Infection, Immunity and Cardiovascular Disease, University of Sheffield, Sheffield, UK; 5 Insigneo Institute for in Silico Medicine, University of Sheffield, Sheffield, UK

**Keywords:** Computational model, Endothelial cells, Particle-based model, Shear stress, Cell migration

## Abstract

Endothelial cells (ECs) play a major role in the healing process following angioplasty to inhibit excessive neointima. This makes the process of EC healing after injury, in particular EC migration in a stented vessel, important for recovery of normal vessel function. In that context, we present a novel particle-based model of EC migration and validate it against in vitro experimental data. We have developed a particle-based model of EC migration under flow conditions in an in vitro vessel with obstacles. Cell movement in the model is a combination of random walks and directed movement along the local flow velocity vector. For model calibration, a set of experimental data for cell migration in a similarly shaped channel has been used. We have calibrated the model for a baseline case of a channel with no obstacles and then applied it to the case of a channel with ridges on the bottom surface, representative of stent strut geometry. We were able to closely reproduce the cell migration speed and angular distribution of their movement relative to the flow direction reported in vitro. The model also reproduces qualitative aspects of EC migration, such as entrapment of cells downstream from the flow-disturbing ridge. The model has the potential, after more extensive in vitro validation, to study the effect of variation in strut spacing and shape, through modification of the local flow, on EC migration. The results of this study support the hypothesis that EC migration is strongly affected by the direction and magnitude of local wall shear stress.

**Electronic supplementary material:**

The online version of this article (10.1007/s10237-019-01239-w) contains supplementary material, which is available to authorized users.

## Introduction

Endothelial cells (ECs) cover the inner surfaces of blood and lymph vessels and are necessary to ensure proper function. Of particular interest are vascular endothelial cells which form a single cell layer on the inside of blood vessels, in contact with the blood. If the layer of ECs is damaged or disrupted, smooth muscle cell (SMC) proliferation from the medial layer of the vessel wall into the lumen may occur. Recovery of ECs following injury is crucial to suppress the proliferation of SMCs (Iqbal et al. 2013; Tahir et al. 2014; Jukema et al. 2012). If inhibition of SMC proliferation is delayed, excessive neointimal tissue growth narrows the lumen, restricting blood flow Jukema et al. (2012).

One particularly important case is the growth of neointimal tissue in a coronary vessel after stenting. Stenting is a frequently used coronary intervention, consisting of the implantation of a metal mesh called a stent into the vessel to restore the lumen, following development of coronary atherosclerosis. However, the stent struts and the balloon are used to deploy it partly or completely remove the endothelium and damage the vessel wall. Multiple studies suggest that endothelial recovery is one of the most important limiting factors in post-stenting coronary healing, and delayed or incomplete recovery can lead to in-stent restenosis or stent thrombosis (Douglas et al. 2013; Iqbal et al. 2013; Chaabane et al. 2013).

Endothelial recovery consists of two main processes: EC migration and proliferation. Studying both processes in detail in vivo is challenging, so to obtain clearer insights into EC behaviour in vitro and ex vivo phantom vessel studies are used. One particular in vitro study setup deals with migration of ECs, with proliferation often suppressed (e.g. by serum starvation) (Tardy et al. 1997; Hsiao et al. 2016; Ostrowski et al. 2014; DePaola et al. 1992; Teichmann et al. 2012). These studies have shown that EC migration is affected by the local flow direction and velocity, but the exact relationship is not yet clear (Hsiao et al. 2016; Ostrowski et al. 2014). The two main hypotheses are that the ECs migrate either in the direction of the local shear force exerted by the flowing liquid, or to the location where the shear is minimized (Tardy et al. 1997; Hsiao et al. 2016). The model discussed in this paper implements the first of these two hypotheses.

One particular experimental condition used to elucidate the process of migration is a flow chamber with a ridge, or multiple ridges, representing stent struts (Hsiao et al. 2016). These ridges disturb the flow, creating zones of backflow and recirculation, and through that disrupting downstream cell migration. This setup reproduces the main features of flow in a stented artery, has a well-defined geometry, and allows continuous imaging of the migrating ECs over several days.

To study endothelial recovery and wound healing in stented vessels, we have developed an in silico model for EC migration under flow conditions. Recirculation zones are a distinguishing feature of stented vessels, so an important goal for the model is to capture EC behaviour in these zones correctly. When such a model is sufficiently validated, it can subsequently be used to study the effects of strut spacing and shape on endothelial recovery.

Most existing models of collective cell migration study the migration under either no flow conditions (e.g. (Kuzmic et al. 2019; Vitorino et al. 2011) and the models reviewed in Scianna et al. 2013), or under conditions of interstitial flow (reviewed, e.g. in Mitchell and King 2013). The reason is that these models are focused on studying de novo formation of blood vessels, often in the context of cancer. This process is significantly different from reendothelization in an existing vessel, where strong blood flow exists and affects EC migration (Teichmann et al. 2012; Shi and Tarbell 2011; Dabagh et al. 2017). The novelty of our model lies in coupling the flow model in a 3D channel to a particle-based model of collective migration on the surface of this channel to study the relation between flow patterns and EC migration.

The in vitro experimental setup described above (Hsiao et al. 2016) was selected to be used as the ground truth against which the in silico EC migration model is validated, due to its ability to provide time-series movement data for EC migration in a well-defined environment. We aim to build a phenomenological in silico model that would help us explore EC migration in various geometries and suggest prospective stent designs to be tested in vitro and in vivo.

Section 2 describes the details of the in silico model, as well as the simulation domain and the method for model calibration based on an in vitro experiment reported in Hsiao et al. (2016). Section 3 describes the application of this model to two other experimental geometries and also presents a comparison between flows of perfusion medium and whole blood in similar geometries. Some discussion of the results is given in Sect. 4, and conclusions are presented in Sect. 5.

## Materials and methods

A particle-based model of EC migration under flow was developed and validated against in vitro experimental data. Here, the formulation of the model, as well as the shape and parameters of the modelled flow system, is described.

### Model formulation

Every single EC is modelled as a particle. These particles interact via a combination of a truncated Lennard-Jones (LJ) 12/6 potential for non-overlapping nearby particles, and a soft-core Neo-Hookean repulsion force for particles that overlap:

1 $$F_{\text{int}} \left( r \right) = \left\{ {\begin{array}{*{20}l} { - 48 \epsilon \left( {\left( {\frac{\sigma }{r}} \right)^{12} - \left( {\frac{\sigma }{r}} \right)^{6} } \right),} \hfill & {2r_{\text{cell}} < r < r_{\text{cutoff}} } \hfill \\ {\frac{{8a^{3} C\left( {16a^{2} - 36\pi ar_{\text{cell}} + 27\pi^{2} r_{\text{cell}}^{2} } \right)}}{{3r_{\text{cell}} \left( {4a - 3\pi r_{\text{cell}} } \right)^{2} }},} \hfill & {r < 2r_{\text{cell}} } \hfill \\ {0,} \hfill & {r > r_{\text{cutoff}} } \hfill \\ \end{array} } \right.$$

where ${F}_\text{int}$ is the interaction force, $r$ is the distance between cells, $\epsilon$ is an interaction constant, $r_{\text{cell}}$ is the radius of a cell, $\sigma = 2r_{\text{cell}}$ is the equilibrium distance, $r_{\text{cutoff}}$ is the maximum interaction distance, $C$ is the elastic constant, and $a$ is the contact area and is approximated by

2 $$a = \sqrt {\frac{{r_{\text{cell}} }}{2} \cdot \left( {2 r_{\text{cell}} - r} \right)}$$

**Fig. 1:**
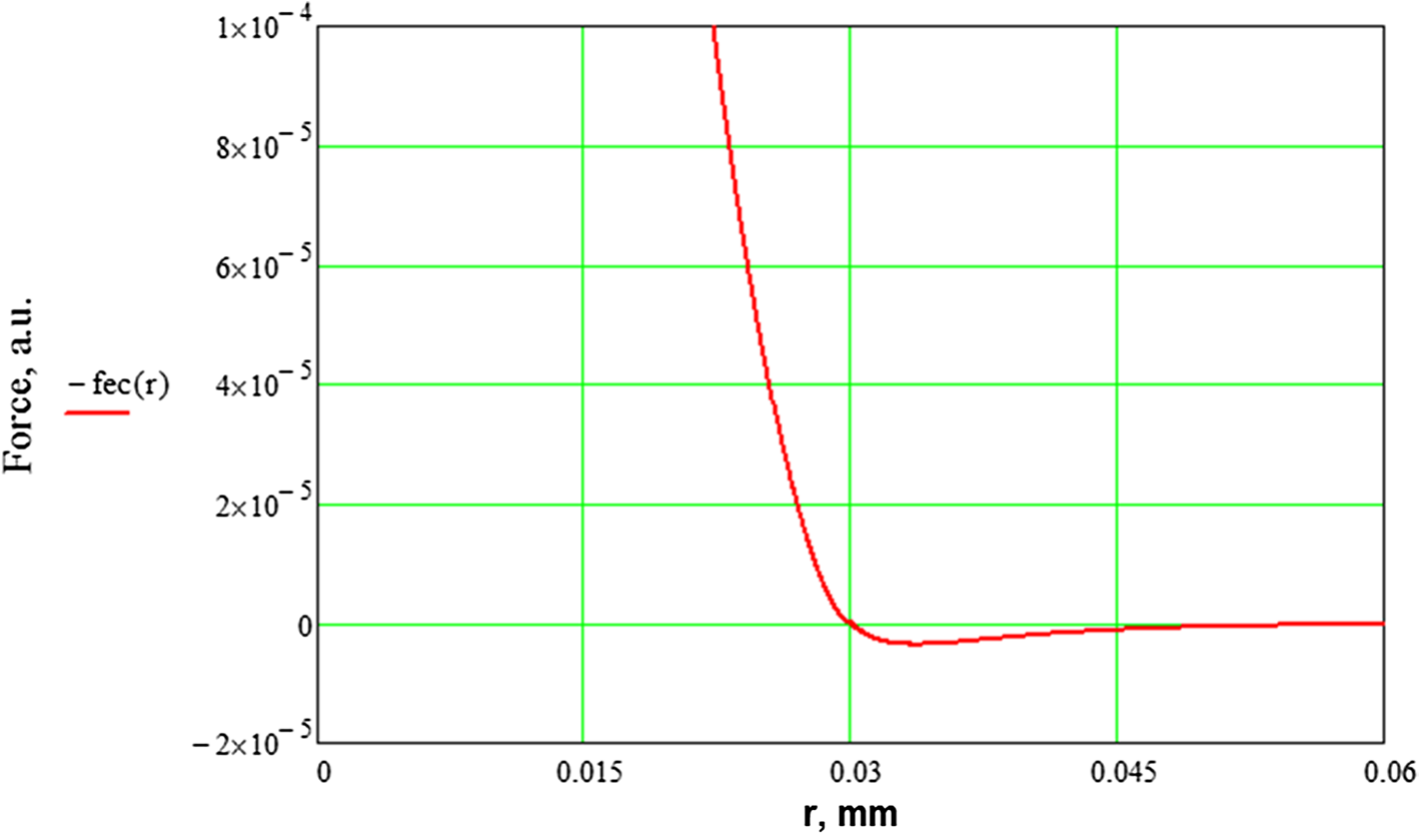
Cell–cell interaction force as function of distance, *r* used in the model. The particle radius is set to 0.015 mm in the model, meaning that cells 0.03 mm apart are touching, but not overlapping. An LJ interaction force is used for distances greater than 0.03, and soft-core repulsion is used for smaller distances (see text). The force is given in terms of arbitrary units (a.u.). For completely overlapping cells, the repulsion force is $1.658 \times 10^{ - 3} \,{\text{a}}.{\text{u}}.$

The Lennard-Jones force is used as a convenient abstraction of cell behaviour. Cells close to each other tend to stay together, and the cells also attach to the substrate and the struts. Soft-core repulsion, instead of LJ force, is used for overlapping cells to avoid unphysiologically high repulsion for strongly overlapping cells. The resulting interaction force is plotted in Fig. 1. Force is given in terms of arbitrary units (a.u.), since the cell–cell interaction model is purely phenomenological and is not based on real values of cell–cell interaction forces. This force prevents the particles from going through one another and keeps them in a single mat.

Individual simulated ECs also migrate, and this migration depends on the local flow direction. Experimental studies suggest that, for flows within the physiological range, flow affects only the direction of cell movement, not its velocity (Hsiao et al. 2016).

It is also known that in the absence of flow, cells on a 2D substrate perform random walks (Lee et al. 1995). Because of this, cell movement is modelled as a persistent random walk in the absence of flow, and the model assumes that as the velocity increases the directionality cue becomes stronger. It should be noted that the local velocity vector near the vessel wall is collinear with the direction of the local wall shear force and also is proportionate to it. Shear force is the value most likely affecting the cells’ migration in vivo, but in the model local flow velocity is used as a substitute.

In more detail, each cell is first assigned a random force $\vec{F}_{\text{rand}}$. It is generated as a sum of three normally distributed components along each coordinate axis (*x*, *y*, and *z*), each with a similar variance $c$ and a mean value of 0, which results in a distribution similar to the Maxwell–Boltzmann distribution for velocity. Then, the force direction under the effect of flow is calculated by taking a weighted average of unit vectors in the direction of $\vec{F}_{\text{rand}}$ and in the local flow velocity direction. The random force is persistent, meaning that once it is assigned to a cell, the random component of the force is kept the same for an extended number of computation steps. Each step, for each particle there is a probability $p_{\text{change}}$ per unit time to replace $\vec{F}_{\text{rand}}^{i}$ with a new force drawn from a similar distribution. This allows us to reproduce the characteristic time scale of the random walks performed by the real cells.

We lack data on the exact relationship between the velocity magnitude and the strength of its contribution to migration direction. In this model, we assume a linear relationship between these variables over the velocity range from zero to $v_{\rm max}$, which is selected to produce a high, but physiological value of WSS. Above $v_{\rm max}$, the weighting remains constant. The weight is then defined as:

3 $$w_{\text{vel}} \left( v \right) = \left\{ {\begin{array}{*{20}l} {\frac{v}{{v_{{\rm max} } }}\left( {w_{{\rm max} } - w_{{\rm min} } } \right) + w_{{\rm min} } ,} \hfill & {v < v_{{\rm max} } } \hfill \\ {w_{{\rm max} } ,} \hfill & {v \ge v_{{\rm max} } } \hfill \\ \end{array} } \right.$$

where $v$ is the local velocity and $w_{{\rm min} }$ and $w_{{\rm max} }$ are the minimum and maximum weights of the velocity direction.

Next, the resulting direction $\vec{d}$ for the migration force is calculated as:

4 $$\vec{d} = \vec{e}_{\text{rand}} \left( {1 - w_{\text{vel}} } \right) + \vec{e}_{\text{vel}} w_{\text{vel}}$$

Here, $\vec{e}_{\text{rand}}$ and $\vec{e}_{\text{vel}}$ are unit vectors in the directions of the random force and the flow velocity, respectively. Then, the resulting migration force $\vec{F}_{\text{migr}}$ is found as:

5 $$\vec{F}_{\text{migr}} = \left| {\vec{F} _{\text{rand}} } \right|\frac{{\vec{d}}}{{\left| {\vec{d}} \right|}}$$

The total force acting on the *i*-th particle equals:

6 $$\vec{F}_{\text{total}}^{i} = \vec{F}_{\text{migr}}^{i} + \mathop \sum \limits_{{j \in Nb^{i} }} \vec{F}_{\text{int}}^{ij}$$

where ${\text{Nb}}^{i}$ is the neighbourhood, or all particles within the cut-off distance from the *i-*th particle.

A simulated cell radius of $r_{\text{cell}} = 15 \,\upmu{\text{m}}$ was selected based on experimental data (Hsiao et al. 2016) as a reasonable approximation of the size of an EC. The movement of cells was solved using an over-damped version of Newton’s second law of motion, and time integration was performed using a fourth-order Runge–Kutta scheme with a variable time step.

The ECs are modelled as spherical particles that adhere to the substrate and do not disturb the flow. The contact surfaces (e.g. substrate and ridges) are modelled as a hexagonal lattice of highly overlapping, fixed, spherical particles of similar size (centres located $1r_{\text{cell}}$ apart), further called *obstacle elements*, to prevent the EC particles from going through the ridges and substrate surfaces.

A lattice Boltzmann (LB) method (Kruger 2017) is used to calculate the flow inside the experimental configurations, and velocity is mapped from the flow domain to the corresponding EC particles. We have used the Palabos code (Latt et al. 2013) for flow simulations (LB lattice cell size $\Delta = 0.015 \,{\text{mm}}$).

### Model calibration

Since the model proposed is rule-based and abstracts away most of the underlying intracellular processes, the model parameters have to be calibrated based on experimental data such as cell trajectories to produce the results in agreement with these experimental results.

The interaction forces are necessary to keep the cells attached to the substrate, to keep the cells from overlapping with each other, and to facilitate cell movement at a realistic speed. Since the aim of this simulation does not include studying mechanical stresses or any effects of these forces on cellular biology, we can formulate the forces in arbitrary units. A set of parameters that results in a realistic average cell migration speed of $50\;\upmu{\text{m}}$ per hour in a flat channel under flow similar to that used in Hsiao et al. (2016) is detailed in Table 1.

**Table 1:** List of model parameters and their values

Parameter	Value	Comment
$r_{\text{cell}}$	$1.5 \times 10^{ - 5} {\text{ m}}$	Based on the experimental values for EC area reported in Yu et al. (1997) and later used in Peirce et al. (2004), Li et al. (2019)
$C$	$0.1$	Source: Tahir et al. (2014)
$\sigma$	$2r_{\text{cell}}$	Equilibrium distance where $F_{\text{int}} = 0$
$\epsilon$	$3 \times 10^{ - 7}$	Selected to minimize the discontinuities in force derivative at $r = \sigma$
$r_{\text{cutoff}}$	$4r_{\text{cell}}$	Interaction force at this distance is less than $10^{ - 6} \;{\text{a}}.{\text{u}}.$
$v_{{\rm max} }$	$0.036\;\frac{\text{m}}{\mathrm{s}}$	Selected based on the flow velocity near the vessel wall
$w_{{\rm min} }$	0.3	
$w_{{\rm max} }$	0.7	
*c*	$9 \times 10^{ - 4}$	

Since for this model we assume a linear relationship between the flow magnitude and the cell’s response over a range from zero to $v_{{\rm max} }$, the area of linear response has to be selected. Based on the flow velocity near the vessel wall, the maximum relevant velocity was selected as $v_{{max} } = 0.036\;\frac{\text{m}}{\mathrm{s}}$. For the values used in the simulation (LB lattice size of $\Delta = 0.015\;{\text{mm}}$ and dynamic viscosity of fluid $\nu = 1 \times 10^{ - 3} \;{\text{Pa}}\;{\text{s)}}$, this translates into a maximum wall shear stress of

7 $$\nu \cdot \frac{{v_{{\rm max} } }}{\Delta } = 10^{ - 3} \cdot \frac{0.036}{{0.015 \times 10^{ - 3} }} = 2.4\;{\text{Pa}} .$$

This WSS lies well within the physiological range for arteries and is considered high enough for normal function of endothelium (Malek et al. 1999). With this, the cells’ response to the flow in the model is governed by $w_{{\rm min} }$ and $w_{{\rm max} }$, while the cells’ persistence is determined by $p_{\text{change}}$. To calibrate $p_{\text{change}}$, cell migration in a flat channel without ridges was simulated using a range of values for $p_{\text{change}}$.

### Ridged channel configuration

**Fig. 2:**
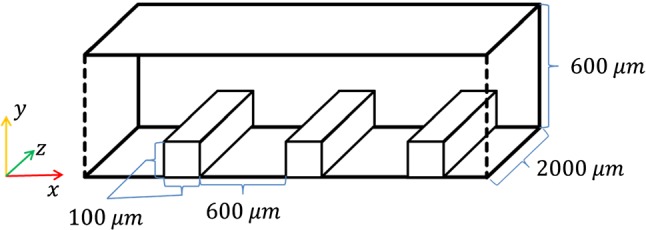
Schematic of the ridged flow chamber used in the simulations. Flow is from left to right. Endothelial cells are seeded in a sheet directly upstream from the leftmost ridge (see text). The flow inlet is 5 mm upstream from the leftmost ridge. Ridge and chamber dimensions are not drawn to scale

A flow chamber, following the in vitro setup (Hsiao et al. 2016), was simulated containing three distinct ridges. The chamber is schematically shown in Fig. 2 (not to scale).To produce a flow and wall shear stress profile in the channel, a parabolic 2D Poiseuille flow is prescribed at the inlet, and a zero-gradient (free flow) boundary condition was defined at the outlet.

Initially, the ECs are seeded on top of the substrate in a hexagonal pattern directly upstream from the first ridge. The cells centres are located $60 \;\upmu{\text{m}}$ or $4r_{\text{cell}}$ apart. Four leftmost rows of ECs (with the lowest $x$ coordinate) are set immobile to prevent cell migration outside of the simulation domain. The typical number of mobile EC particles in the simulation domain is around 3000.

The simulated channel differs from the in vitro channel in that it is narrower (in the *z*-direction) to reduce computational costs. On the sides of the channel, the ECs are contained by frictionless force walls. Also, cell movement is restricted to a narrow zone near the substrate and the struts, reflecting the tendency of real ECs to stay in a monolayer. This is done by restricting cell centre positions to within $3r_{\text{cell}}$ away from obstacle elements. Also, following the in vitro experiment, a similar channel without ridges was used as an alternative geometry to study cell migration in an undisturbed flow.

### Backward step configuration

**Fig. 3:**
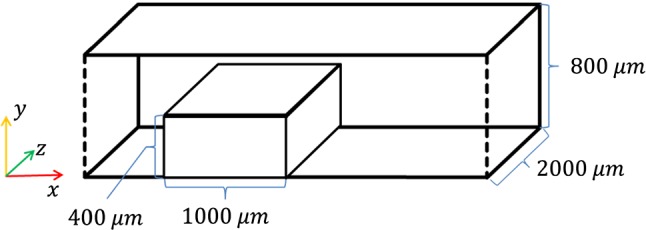
Schematic of the backward step flow chamber used in the simulations. Flow is from left to right. Endothelial cells are seeded in a sheet directly downstream from the step (see text).The flow inlet is 2 mm upstream from the left side of the step. Step and chamber dimensions are not drawn to scale

The model was also applied, without any changes, to a geometry reported in an earlier study of EC migration (Tardy et al. 1997) in a channel with a backward-facing step, with cells seeded downstream of the step (see Fig. 3). In this study, the authors hypothesized that cells migrate towards the location where shear is minimized. Applying the model to this setup aims to determine whether migration in the direction of the local flow velocity (as opposed to the direction of its gradient, as suggested in Tardy et al. 1997) can explain the behaviour observed in this experiment. Simulation of cell migration started from the initial configuration of the monolayer for 48 h (similar to the experiment).

The performance of the model was analyzed through comparison between the distribution of cells within the simulation and video data recorded experimentally as reported in Tardy et al. (1997).

### ROCK inhibition in the ridged channel configuration

Since EC migration is inhibited in stented vessels due to reversed cues from the flow downstream from the struts, various approaches have been considered to reduce the extent to which cells follow these cues. One mechanism is to pharmacologically reduce the activity of Rho-associated protein kinase (ROCK) in these cells. ROCK controls the migration cascade, and its inhibition has been shown to enhance endothelial migration in a ridged channel in vitro (Hsiao et al. 2016). We aimed to reproduce this effect in silico using the assumption that inhibiting ROCK reduces the contribution of flow cues to the cells’ motion. It is assumed that ROCK inhibition downregulates the cells’ response to weak cues in the low flow area, while strong cues are assumed to be sufficient to activate the cell migration cascade regardless of inhibition. This was achieved by repeating the analyses undertaken in the ridged channel geometry as described in Sect. 2.3 and reducing $w_{{\rm min} }$ from its original value of 0.3 to a value of 0.05.

The performance of the model was analyzed through comparison between the angular distribution of cell movement using cell tracks over a 24-h period (Hsiao et al. 2016).

### Cell-based flow

One notable difference between in vitro and in vivo setups is that in vitro experimental setups are perfused with culture medium, while in vivo whole blood flows through the stented vessel. Unlike culture medium, whole blood contains red blood cells, platelets, and other, less numerous, types of cells. This difference in composition gives rise to differences in viscosity and changes in the local flow patterns and shear stresses acting on the ECs, especially at the microscale. To assess if these differences are relevant for the ridged channel geometry studied here, we performed a 2D simulation of cell-based blood flow in a similar ridged channel. An LB model of flowing plasma with particles suspended in it was used for the cell-based flow simulation. The model is two-way coupled, and the cells suspended in the plasma affect the flow. The immersed boundary method is used, which is a fluid–structure interaction method, so the RBC membrane exerts a force on the fluid and the RBC membrane feels the velocity of the fluid. The model implementation is described in detail in Czaja et al. (2018).

## Results

### Model calibration

**Fig. 4:**
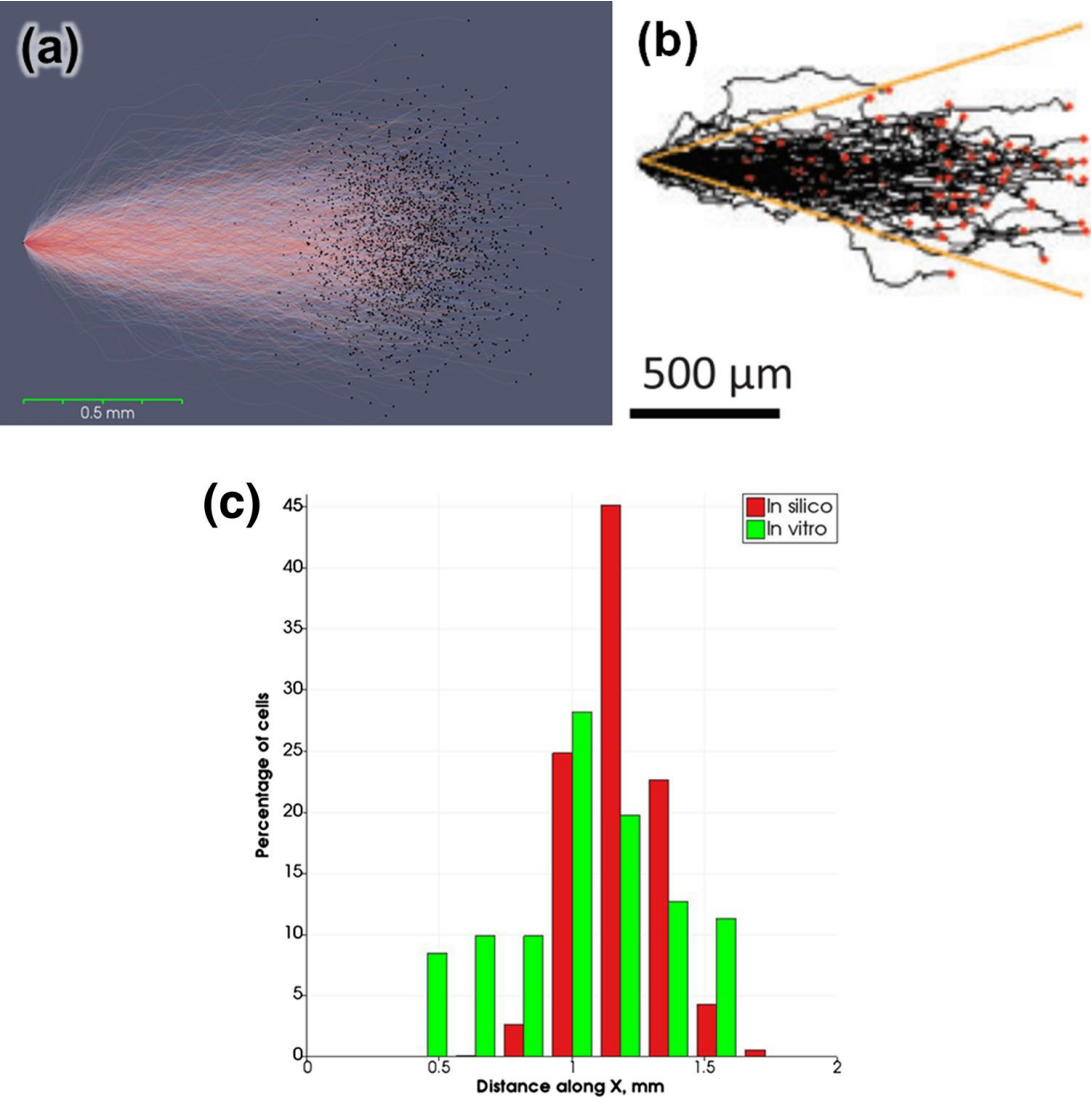
Cell trajectories over 24 h for **a** in silico and **b** in vitro [adapted from Hsiao et al. (2016)] experiments. Dots are the final positions of cells. In **a**, each cell’s track is assigned its own colour between red and blue; **c** axial distribution of cells in a flat channel in silico (red) and in vitro (green)

The best agreement for cell movement speed and the cell trajectory angular distribution between the model and cell migration in a flat channel without ridges was achieved for $p_{\text{change}} = 0.075\,{\text{per}}\,{\text{hour}}$. Cell trajectories over 24 h are shown in Fig. 4, for the (a) in silico and (b) in vitro (adapted from Hsiao et al. 2016) cases, and Fig. 4c shows the normalized axial distribution of cells along the X axis for both cases. A comparison of angular distributions of cell trajectories is presented in 3.2.

### Ridged channel configuration

**Fig. 5:**
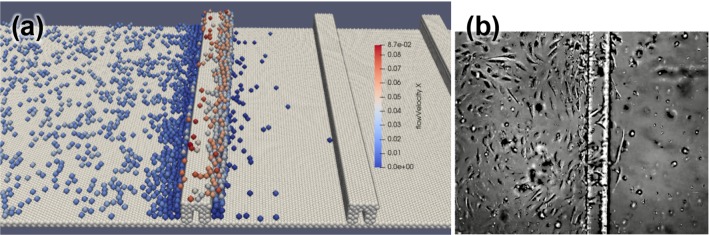
**a** Simulated cells migrating downstream over the first ridge. Individual cells are coloured by the local flow velocity component along the *X* axis. Cells get trapped when they enter a disturbed flow zone downstream from the ridge (deep blue cells). See also Supplementary video 1; **b** in vitro migration of cells over a ridge Adapted from Hsiao et al. (2016)

Next, the model was applied to the case of EC migration in a ridged channel. The model was able to replicate cell entrapment in recirculation zones behind the struts, see Fig. 5 and Supplementary video 1. If the simulation is continued after 24 h (the time over which the cell trajectories were tracked in vitro), ECs eventually start crossing the second ridge. It should be noted that this behaviour is different from what is observed in vitro: real cells change their phenotype after about 40 h, start forming a confluent monolayer, and stop migrating over the ridge (Hsiao et al. 2016). This phenotype change is, however, currently not part of the in silico model, as it focuses on the migration of ECs.

**Fig. 6:**
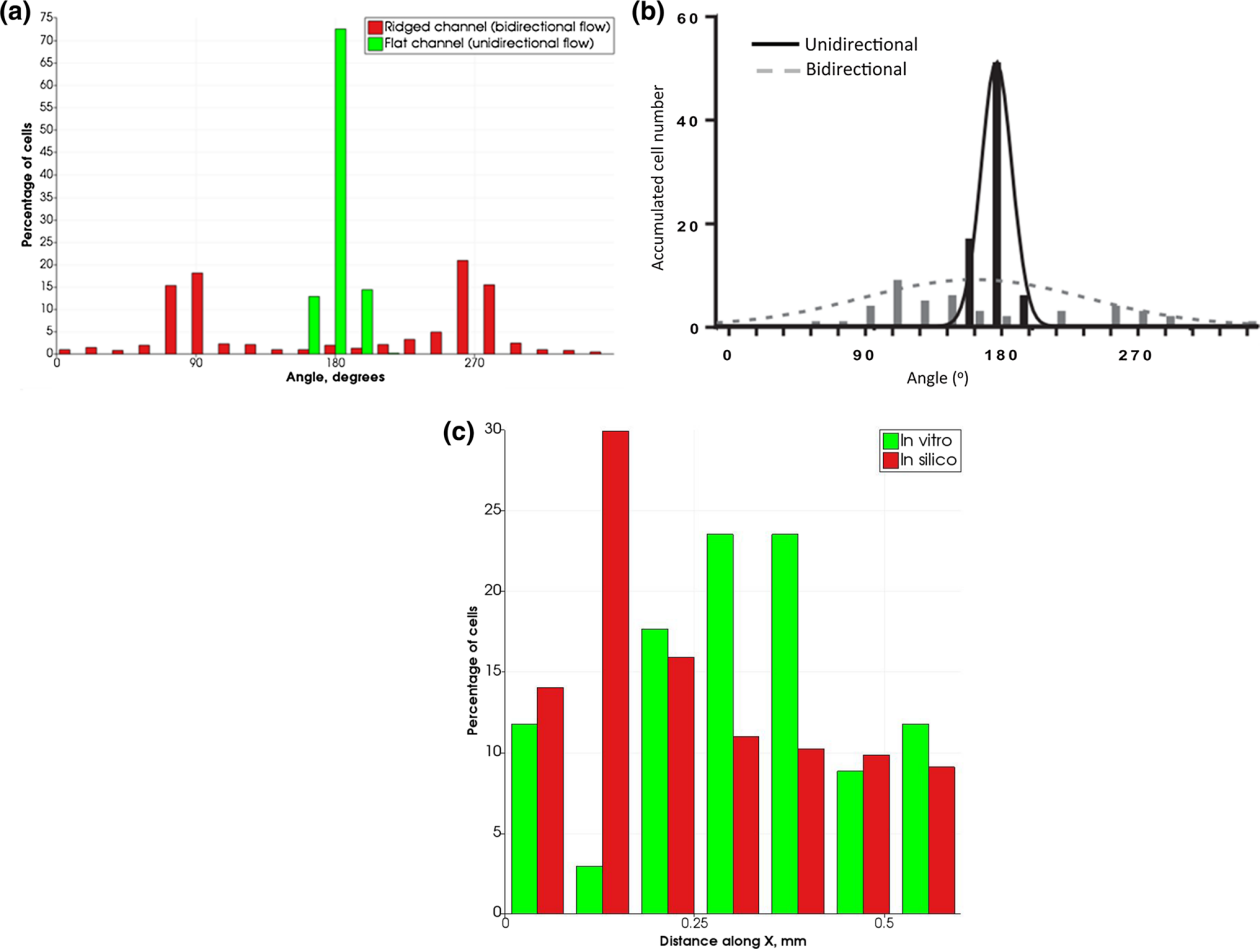
Angular and axial distributions of cell displacement over 24 h. **a** Angular percentage distribution for the in silico experiment. **b** Total number of cells for similar angles for in vitro experiment. Plots show the migration for flat and ridged channels. In vitro distribution plot adapted from Hsiao et al. (2016). 180° correspond to the downstream flow direction. **c** Axial distribution of cells in a ridged channel in silico (red) and in vitro as reported in Hsiao et al. (2016) (green) For the ridged channel, only cells downstream from the ridge are considered, and zero coordinate is located $50\,\upmu{\text{m}}$ downstream from the ridge

Following the in vitro data presented in Hsiao et al. (2016), angular distributions of cell movement were compared using cell tracks over 24 h. Figure 6 shows the distributions for the in silico (a) and in vitro (b) migration for flat and ridged channels. For the ridged channel, the tracks downstream from the ridge were considered, and the coordinate system origin was assumed to be $50\,\upmu{\text{m}}$ downstream of the ridge.

### Backward step configuration

**Fig. 7:**
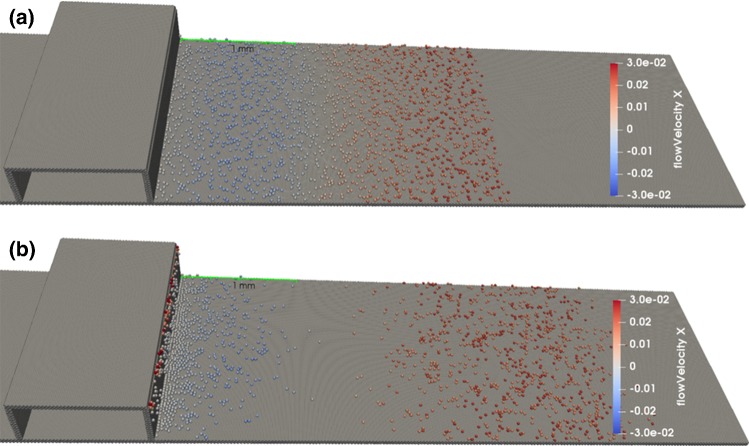
Cell migration in a flow chamber similar to the one reported in Tardy et al. (1997). **a** Starting configuration; **b** ECs after 48 h of migration. See also Supplementary video 2

The experimental data reported in Tardy et al. (1997) showed that cells next to the obstacle move closer to it and form a denser monolayer, while the remaining cells migrate downstream. After simulating cell migration from the initial monolayer for 48 h the model produced qualitatively similar results (Fig. 7, Supplementary video 2), but the zone with low cell density was further away from the obstacle in the model than in the experiment. In the in silico model, the lowest density was observed about 1.2 mm downstream from the ridge, while the in vitro experiment the lowest cell density is found approximately 0.8 mm downstream.

### ROCK inhibition in the ridged channel configuration

**Fig. 8:**
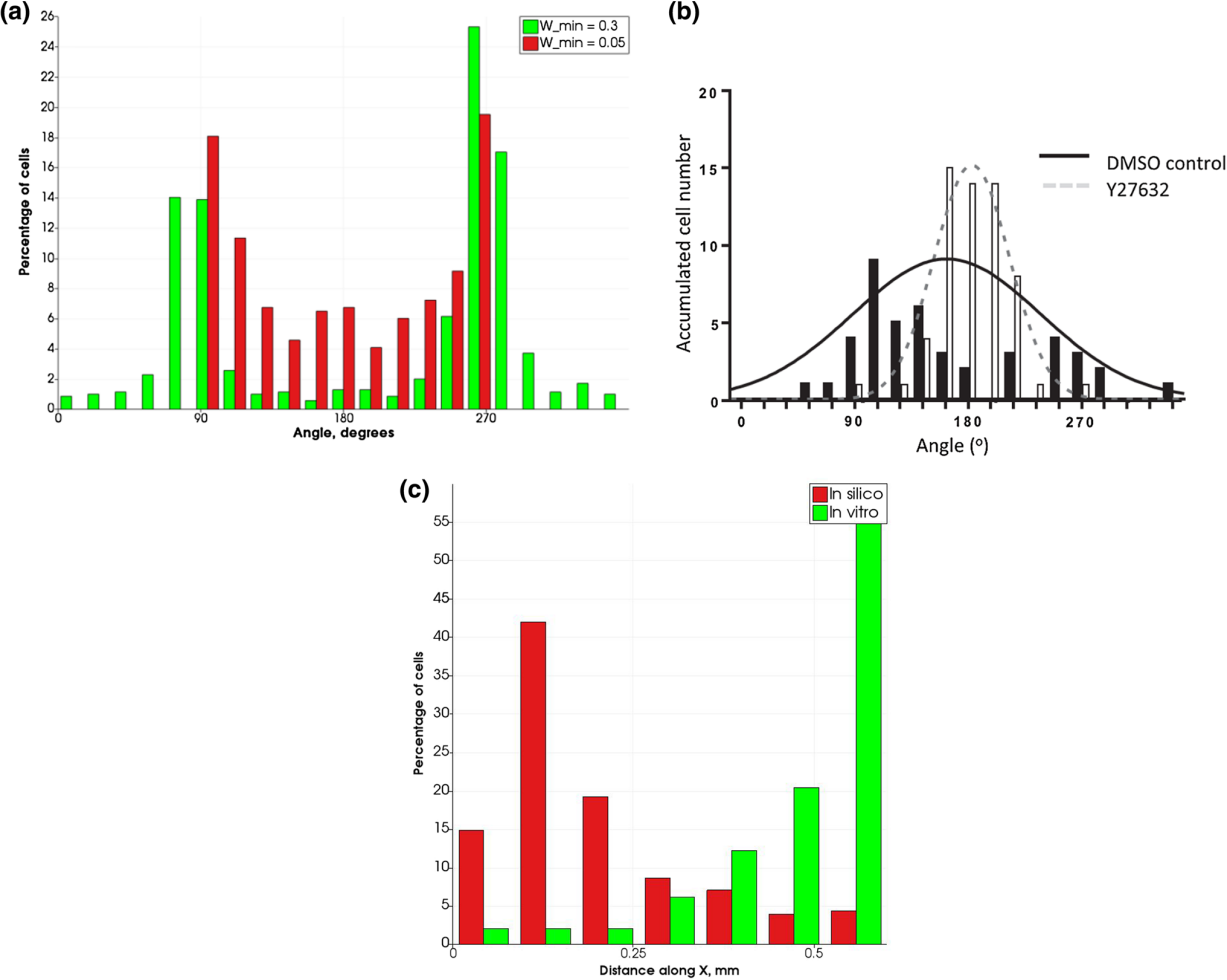
Angular and axial distributions of cell displacement for inhibited cues from the flow. **a** Angular percentage distribution for the in silico experiment for cases of inhibited and non-inhibited cells. **b** Total number of cells for similar angles for in vitro experiment in a ridged channel, for pharmacologically inhibited ROCK $(2\,\upmu{\text{M}}\,{\text{Y27632)}}$ and for non-treated controls (same as bidirectional plot in Fig. 5b). In vitro distribution plot adapted from Hsiao et al. (2016). 180° correspond to the downstream flow direction. **c** Axial distribution of cells in silico (red) and in vitro as reported in Hsiao et al. (2016) (green). For the ridged channel, only cells downstream from the ridge are considered, and zero coordinate is located $50\,\upmu{\text{m}}$ downstream from the ridge

Figure 8 shows the angular distributions of cell displacement for inhibited cues from the flow for $w_{{\rm min} } = 0.05$ (a) and the experimental data from Hsiao et al. (2016) for cells pharmacologically treated to inhibit ROCK.

### Cell-based flow

**Fig. 9:**
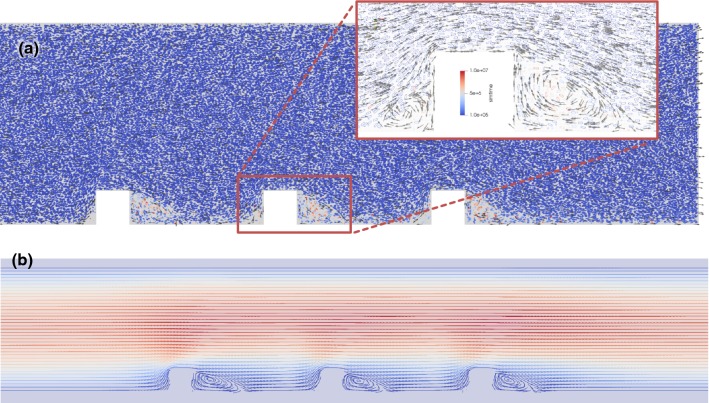
**a** Snapshot of 2D cell-based flow simulation. Flow from left to right. Outlines of individual blood cells are shown. The cells are coloured by their time of residence, blue is fast moving, and red ones stay in the same area. Recirculation zones with reduced cell content are visible after both ridges and also in front of the ridges, but smaller. **b** Flow lines for cell-free liquid used in Sects. 3.1–3.4 in similar geometry, where red colour indicates faster flow and blue indicates a slower one

The simulation results, shown in Fig. 9a, show the recirculation zones for cell-based flow with Reynolds number $\text{Re} = 210$. Figure 9b shows flow lines for the cell-free flow used in Sects. 3.1–3.4. The recirculation zones are located similarly, with the recirculation zones being somewhat smaller for the cell-based flow. This supports the claim that the in vitro flow setup can adequately capture the features of flow in real arteries. However, the different concentrations of red blood cells along the wall of an in vivo blood vessel may affect the WSS distribution compared to the cell-free case. We have not further analyzed these findings in detail.

## Discussion

In this study, we propose a novel particle-based model of EC migration and test it against the in vitro experimental results. We aim to model the cell behaviour, governed by complex biochemistry and mechanotransduction, as a few simple rules, and to build a model that can be used to study cell migration in geometries corresponding to various in vitro and in vivo vessels. There exist some very complex models for EC behaviour, e.g. Dabagh et al. (2017), but they cannot be used to study large-scale collective behaviour. There are also multiple continuous models of EC migration (Kuzmic et al. 2019; Scianna et al. 2013), but they usually consider migration in the absence of flow, where the cells move in the direction of growth factor gradient, and are aimed at simulating de novo arteriogenesis.

Our model is able to reproduce the main features of EC migration in vitro under flow conditions (Hsiao et al. 2016), such as migration downstream in a flow channel, cells moving across ridges, and cell entrapment downstream from the ridges. When a flow velocity matching the experimental case is prescribed, the model is able to reproduce the angular distribution of cell movement and the average migration speed. These values are in a good agreement with the experimental data for the cases of ridged and flat channels, while there are some differences in the axial distributions, with in vitro cell distributions having less pronounced peaks.

When applied to the geometry from a different in vitro experiment (Tardy et al. 1997), the model was able to reproduce the qualitative behaviour of ECs studied there, which were seeded downstream from an obstacle. The cells close to the ridge (in the region of reversed flow) migrated upstream even closer to the ridge, while the cells further from the ridge migrated further downstream. Some simulated cells even moved from the bottom of the channel to the side surface of the ridge. It is unclear if this behaviour was observed in vitro to any extent, since in the experimental paper only the cells at the bottom of the channel were reported. This suggests that the movement reported in that paper might have occurred in the direction of the local shear stress, and not of the local shear stress gradient, as was suggested by the authors of the experimental study in Tardy et al. (1997). The differences in the position of the area with the lowest cell density can be attributed to the differences between flow patterns in silico and in vitro, or by the in vitro experiment using a different substrate than what the model has been calibrated for.

However, this model relies on several assumptions, two of which are particularly important. First of all, the flat shape of ECs is completely ignored, and instead ECs are represented as point particles with spherically symmetrical interaction laws. This difference in shape may be responsible for some of the differences between the in silico and in vitro axial distributions: while round in silico particles can gather in high concentrations, real flat ECs have to spread out over a larger area, making the concentration peak less pronounced.

Second, the interaction between the magnitude of flow and the skewedness of cell movement is most likely not linear; however, more data are required to determine the exact nature of this relationship. The model includes multiple parameters that have little basis in cellular biology, but most of them affect the migratory behaviour only to a small extent: for example, the exact shape of cell–cell interaction potential is irrelevant, as long as it keeps the cells attached to the substrate and prevents the cells from aggregating.

The in vitro model used for validation provides very detailed data for cell migration under flow conditions. We were able to demonstrate that the in silico model is able to match quantitative parameters of the cell migration in vitro, such as average movement speed and angular distribution of trajectories. This allows us to consider the model rather validated for the case of untreated cells.

However, the agreement between simulation of ROCK-inhibited cells and experimental observations is not as clear as the in silico cells still tend to move perpendicularly to the flow direction after ROCK is inhibited (a mechanism that translates into reducing $w_{{\rm min} }$ in the model). This might be due to the attraction between the cell particles and the obstacles that make up the ridge in silico. This perpendicular movement results in the simulated cells travelling a much smaller distance downstream than in vitro. Another set of simulations (omitted from the paper) also shows that angular distribution produced by the model is rather insensitive to lowering $w_{{\rm max} }$ in the range from 0.7 to 0.45. For $w_{{\rm min} }$, no such study was performed due to the computational costs involved in setting up multiple simulations. The decreased value of $w_{{\rm min} }$ (0.05) is arbitrary, but this response mechanism is based on what is suggested by the currently available experimental results. As the in silico model does not currently include details of cell biology, it is incapable of capturing features such as phenotype change and corresponding changes in behaviour. The simulations show that the response of cells to ROCK inhibition is apparently more complex than has been assumed in the model, and more experimental data together with a more sophisticated model are required to study the effects of pharmacological intervention on cell migration.

On the other hand, the model gives a reasonable prediction for the behaviour of untreated ECs and should provide a reasonable scenario of endothelium regeneration for a bare metal stent (BMS). This allows it to be used as a part of multiscale models of restenosis in BMS, such as the one described in Zun et al. (2017, 2019).

In vitro EC migration models are useful for model calibration and validation as conditions between different experiments are controlled as much as possible. However, the exact behaviour of the cell in vitro depends on the particular setup used in the experiment: for example, using different substrates for in vitro monolayer can significantly affect the cells’ reaction to flow (Teichmann et al. 2012), and the number of passages since the cells were collected from a live organism can also influence cell behaviour (Timraz et al. 2016). Translating the results from in vitro observations to predict cell behaviour in vivo poses additional challenges as effects such as the influence of transmural pressure (DeMaio et al. 2004) and the influence of different cell types forming a three-dimensional structure in the vessel wall in the full three-dimensional in vivo geometry (Stegemann and Nerem 2003). As a result, the current model will provide only qualitative insights into endothelial regeneration in vivo based on these in vitro calibration and validation steps.

Nevertheless, the model can, after a more extensive validation on new in vitro data, be used for screening candidate stent design approaches in terms of variation in strut spacing and shape, along with resulting flow patterns, on EC migration. Following in silico screening, the most successful designs can then be subjected to in vitro testing prior to incorporation into prospective novel stent designs for in vivo assessment.

## Conclusions

A novel particle-based model of EC migration is presented. It has been tuned to reproduce the behaviour found in experiments in vitro. The results of this study support the hypothesis that EC movement is strongly affected by local wall shear stress direction and magnitude, and also explain the behaviour reported in an earlier work, where the authors attributed cell migration to minimization of shear stress gradient. Our results show that this hypothesis is not required to reproduce in vitro observations of cell migration.

## Electronic supplementary material

Below is the link to the electronic supplementary material.

Supplementary material 1 (AVI 11197 kb)

Supplementary material 2 (AVI 1427 kb)
